# *Halomonas* sp for sustainable agriculture a potential halo bio fertilizer for tomato plants with biocontrol activity against *Fusarium* wilt under saline environments

**DOI:** 10.1038/s41598-025-12974-9

**Published:** 2025-08-21

**Authors:** Ahmed Ahmed Abdelmonaem Mousa, Wafaa Hanafy Mahmoud, Hosam Easa Elsaied, Adel Elsayed Elbeltagy

**Affiliations:** 1 Botany Department, Faculty of Agriculture, Menoufia University, Shibin El Kom, Egypt; 2 Department of Genetics and Genetic Engineering, National Institute of Oceanography and Fisheries, NIOF, Cairo, Egypt

## Abstract

Halophilic bacteria are remarkable microorganisms that excel in hypersaline environments. Their significant potential in various fields, such as industry and agriculture, positions them as vital players in advancing our technological and ecological efforts. In this study, three bacterial strains (QSLA1, QSLA2, and QSLA3) were successfully isolated from solar saltern ponds, attached to Qarun Lake, Fayoum governorate, Egypt, using nutrient agar (NA) culture medium derived from pond water. Morphological and physiological characterization revealed that these isolates are rod-shaped, gram-negative, catalase-positive, and motile. All isolates were identified as not spore-forming bacteria. The halo tolerance assay demonstrated that QSLA1 and QSLA2 are extremely halophilic, whereas QSLA3 is classified as moderately halophilic. Through 16S rRNA sequence analysis, it was determined that QSLA1 shares 91.26% similarity with *Halomonas* sp. RS-17, while QSLA2 exhibits 96.6% similarity with *Halomonas* sp. strain LR2-3. QSLA3 shows even greater similarity at 97.33% to *Halomonas* sp. GQ30. All isolates are capable of producing indole-3-acetic acid (IAA), but only QSLA2 has the ability to fix atmospheric nitrogen and solubilize insoluble phosphate. Additionally, QSLA1 demonstrates antifungal activity against *Fusarium oxysporium f.sp. lycopersici* in vitro under saline environment. Given these promising traits, we explored the potential of QSLA1 as a bio-control agent under greenhouse conditions at 1.5% salinity. These findings suggest that these bacterial strains could be used to develop sustainable agricultural practices, enhancing crop yields and reducing the reliance on chemical fertilizers and pesticides. Future applications of these strains could provide a valuable solution for improving agricultural productivity in saline environments.

## Introduction

Hypersaline ecosystems are dynamic and diverse habitats that include various terrestrial lakes and deep-sea basins, exhibiting salt concentrations that exceed three times that of seawater, reaching saturation levels. These habitats are effectively categorized into two main types: thalassohaline and athalassohaline waters^39^. Solar salterns, widely distributed across arid and semi-arid regions, can be found at sea level, either as natural formations or as human-made innovations^60^.

Qarun Lake, strategically located in the northern region of Fayoum Governorate, is characterized by its saline, turbid waters and lack of surface outflow^12^. The solar salterns surrounding Qarun Lake feature a well-structured multi-pond system that is interconnected. These ponds are actively utilized by local companies for the efficient production of salt and minerals.

Salinity stress significantly challenges agriculture by reducing productivity, limiting arable land, and causing ionic and osmotic imbalances in plants, which disrupts physiological processes and leads to soil degradation. However, halophilic/halotolerant plant growth-promoting bacteria (PGPB) provide sustainable solutions to mitigate saline stress and enhance plant growth due to their ability to adapt to extreme environments through mechanisms such as ion homeostasis, osmotic balance, and production of compatible solutes^25^.

Halophiles, the remarkable salt-loving microorganisms that thrive in saline environments, are integral components of hypersaline ecosystems. They span all three domains of life: Archaea, Bacteria, and Eukarya^12^. These halophilic bacteria present significant opportunities in agriculture, where they can be employed for bio-control of phytopathogens, the solubilization of essential nutrients, and the stimulation of plant growth through the production of beneficial growth factors^37^.

Tomato fruits (*Lycopersicon esculentum*) rank among the most popular and widely consumed vegetables globally, especially in Egypt^3^. However, tomato plants face challenges from various fungal diseases, with *Fusarium* wilt being one of the most critical. This disease caused by the soil-borne fungus *Fusarium oxysporium f.sp. lycopersici* (Fol), can lead to significant yield losses in both greenhouse and field conditions^53^. In Egypt, *Fusarium* wilt can reduce yields by as much as 25%^1^. The pathogen infiltrates the plant through the roots, colonizes the vascular tissues, clogs the xylem, and induces water stress, which manifests as wilt-like symptoms^53^.

Research by^63^ has demonstrated the effectiveness of moderately halophilic bacteria, particularly *Bacillus subtilis* J9 and *Halomonas* sp. K2-5, in managing stem canker in greenhouse tomatoes. *Halomonas* sp., isolated from a saline habitat in northeastern Algeria, has shown a broad spectrum of antifungal activity against pathogens such as *Fusarium oxysporium, Botrytis cinerea, Phytophthora capsici,* and *F. verticillioides*^35^.

In this study, halophilic bacterial strains were isolated from the solar saltern ponds of a local salt and mineral company. The strains were rigorously tested for their potential to enhance plant growth and exhibit antimicrobial activities against plant pathogens. We meticulously assessed the impact of the isolate QSLA1 on Fusarium wilt disease as well as its effects on the growth of tomato seedlings under saline conditions in controlled greenhouse environments.

## Materials and methods

### Bacterial isolates

In September 2019, water samples were collected from solar saltern ponds, ensuring a comprehensive analysis of their properties. After filtering to remove impurities, the salt concentration was accurately assessed using a refractometer and pH levels were determined with precision using a pH meter. Ten liters of water were gathered from each pond in sterile plastic jars, which were meticulously placed in ice packs for transportation to the biotechnology laboratory in the Botany Department at Menoufia University.

To isolate bacteria, we employed sterilized Nutrient Agar (NA) medium prepared using pond water as follow: 5 gm peptone, 3 gm yeast extract, 15 gm agar agar, and 1 L pond water. A 100 µl aliquot of each water sample was streaked onto NA plates and incubated at 30 °C for periods ranging from 7 to 30 days^5^. Subsequent streaking and sub-culturing were conducted on NA culture medium prepared from the pond water, successfully purifying the isolates, which were then preserved in 30% glycerol and stored in a freezer for future analysis.

### Morphological and physiological characterization of the isolates

The colonies that developed on the plates underwent thorough morphological examination, focusing on their shape, pigmentation, elevation, and optical properties. Additional parameters including shape analysis, gram staining, endospore formation^23^ were rigorously tested as well as bacterial motility using the hanging drop method^20^, and catalase activity^61^.

### Salinity tolerance of the isolates

The bacterial isolates were effectively screened for salt tolerance by using a NA culture medium supplemented with varying concentrations of NaCl (0%, 7%, 12.5%, 20%, and 22%). The plates were incubated for 7 days at 30 °C, and the growth results were meticulously recorded^43^.

### Molecular identification of the isolates via 16S rDNA

#### DNA extraction and polymerase chain reaction (PCR)

Genomic DNA was extracted from bacterial cells cultured aerobically in nutrient broth^7^. The extracted DNA was purified, visualized under UV light with ethidium bromide staining^48^, and securely stored at −20 °C until needed^38^. Utilizing bacterial primers 27F and 1492R, as specified in Table 1, the 16S rRNA gene sequence was successfully amplified using a model PTC-100 thermal cycler (MJ Research Inc., USA)^45^. The presence of amplified products was confirmed by applying 7 μl of the PCR product onto a 1% agarose gel in 1X TAE buffer containing ethidium bromide, and results were visualized with a gel documentation system (Bio-Rad Laboratories)^48^. The PCR products were purified using the QIA quick PCR purification kit protocol (Qiagen) and promptly sent for sequencing.

**Table 1:** Primers used for PCR 16S rRNA sequencing analysis^59^.

Primer name	Orientation	Priming site	Sequence (5´–3´)
27F	Forward	8–27	AGAGTTTGATCCTGGCTCAG
1492R	Reverse	1492–1513	GGTTACCTTGTTACGACTT

#### 16S rRNA sequencing

The sequencing was expertly conducted by Colors Laboratories at El-Etihad Square in Maadi, Cairo, Egypt, following rigorous laboratory protocols. The sequences were thoroughly edited using the Complete Deletion option to eliminate all gaps, utilizing CHROMAS PRO software, version 1.5.

#### Evolutionary relationships of taxa

The resultant 16S rRNA gene sequences were successfully compared with those available in the GenBank databases using the highly effective Basic Local Alignment Search Tool (BLAST) on the National Center for Biotechnology Information (NCBI) website (http://www.ncbi.nih.gov). For alignment, CLUSTAL W 1.6 software was employed^2^, ensuring precise and reliable results. The evolutionary history was robustly inferred using the Neighbor-Joining method^47^. Evolutionary analyses were carried out with confidence using MEGA X^29^. The sequences were meticulously aligned using the embedded MUSCLE algorithm, and the resulting output was leveraged to construct a phylogenetic tree by calculating distance matrices for Neighbor-Joining (NJ) analysis.

#### Evaluation of plant growth under laboratory conditions

The isolates were rigorously evaluated for their growth-promoting properties, with all analyses conducted in duplicate to ensure accuracy. The inoculum for screening was expertly prepared by cultivating halo-bacteria in 20 ml of nutrient broth culture medium, enriched with a 12.5% salt concentration, and subjected to shaking at 120 rpm for three days.

#### Nitrogen fixation test

The capability of bacterial isolates to fix free nitrogen was thoroughly tested using Ashby-free N-agar medium^27^. Bacteria that successfully grew in this medium are unequivocally identified as nitrogen-fixing organisms^26^.

#### Phosphate solubilization assay

The potential of isolates to solubilize inorganic phosphate was determined using the disk diffusion method on a modified Pikovskaya agar culture medium^62^. Colonies of phosphate-solubilizing bacteria (PSB) were clearly identified by the distinct diffusion zones surrounding them, as highlighted by^26^.

#### Indole acetic acid (iaa) production assay

Isolates were confidently screened for IAA production utilizing a refined quantification method^19^. The production of IAA by bacterial isolates was clearly indicated by a distinct pink color change upon the addition of Salkowski’s reagent, as illustrated in Fig. 3. The IAA concentration was accurately estimated against a well-prepared standard curve derived from various concentrations of IAA (Sigma-Aldrich, Germany)^51^.

#### Antagonistic activity of the isolates against plant pathogens

The antifungal activity of bacterial isolates was rigorously assessed through co-cultivation of macro-colonies against key pathogens, including *Fusarium oxysporium f.sp. lycopersici*, a well-known cause of tomato vascular wilt disease^53^; *Alternaria solani*, responsible for early blight in tomato and potato plants^21^; and *Botryodiplodia theobromae*, which leads to die-back and stem-end rot in mango fruits^33^. For this assessment, the pathogens were strategically inoculated onto NA culture medium (with a concentration of 12.5% NaCl) at the center of the plate, while the halophilic bacteria inoculated around the fungus. The antagonistic properties of the bacteria were determined by measuring the zones of inhibited fungal growth, while NA plates inoculated solely with the pathogens served as an effective negative control. All cultures were incubated at a consistent temperature of 25 °C for 7 days^42^.

Also an impactful evaluation of the antibacterial activity of the isolates against *Ralstonia solanacearum* NRRL B-3211, a notorious cause of bacterial wilt affecting a broad spectrum of host plants^41^, was conducted using the well-established disc diffusion method^44^.

#### Determination of bioactive chemical constituents produced by QSLA1 isolate using GC–MS analysis

The chemical compounds produced by the selected isolate QSLA1 were thoroughly evaluated and analyzed using Gas Chromatography-Mass Spectrometry (GC–MS). Extraction of bioactive compounds was executed following the liquid–liquid extraction protocol^14^. Briefly, an aliquot of 5 mL sample was placed in 15-mL centrifuge tubes, and a mixture of extraction solvent (0.29 mL chloroform in 0.28 mL acetone) was introduced rapidly into the sample. The extractant was dispersed into the sample solution via vortexing for 0.5 min resulting in the formation of a cloudy solution (water/acetone/chloroform). The analytes were extracted into the fine droplets of chloroform**.** Centrifugation was applied to separate the two immiscible layers at 4400 rpm for 3 min. The segmented organic layer (chloroform) was thereafter quantitatively transferred into a 2-mL vial and evaporated to dryness at 60 °C. Thereafter, the residue was reconstituted in 1 mL mobile phase and vortexed prior to injection into the GC MS.

#### Gas chromatography–mass spectrometry (GC–MS) analysis

The chemical composition of samples performed using GC-TSQ mass spectrometer (Thermo Scientific, Austin, TX, USA) with a direct capillary column TG–5MS (30 m × 0.25 mm × 0.25 µm film thickness). The column oven temperature was initially held at 60 °C and then increased by 5 °C/min to 250 °C withhold 2 min then increased to 300 with 30 °C/min. The injector temperature was kept at 270 °C. Helium was used as a carrier gas at a constant flow rate of 1 ml/min. The solvent delay was 4 min and diluted samples of 1 µl were injected automatically using Autosampler AS3000 coupled with GC in the split mode. EI mass spectra were collected at 70 eV ionization voltages over the range of m/z 50–650 in full scan mode. The ion source and transfer line were set at 200 °C and 280 °C respectively. The components were identified by comparison of their mass spectra with those of WILEY 09 and NIST14 mass spectral database^24^.

The comprehensive chemical composition of metabolites extracted was detected using a GC–TSQ mass spectrometer (Thermo Scientific, Austin, TX, USA) with a direct capillary column TG–5MS (30 m × 0.25 mm × 0.25 µm film thickness), as detailed by^6^. The percentage composition of each compound was diligently calculated as the ratio of the peak area to the total chromatographic area, with GC–MS peaks confidently assigned through comparison with established data, achieving similarity percentages from the Wiley 275 libraries^31^.

### Controlling tomato *Fusarium* wilt pathogen using QSLA1 (*Halomonas* sp.) under greenhouse conditions

#### Preparation of fungal inoculum

Tomato seedlings (halophyte strain 023) that were 35 days old were utilized for the study. The fungal inoculum was prepared as follows: The fungal strain *Fusarium oxysporium subsp. lycopersici* was cultivated on sterilized barley grain medium, consisting of 100 g of washed, dried barley grains mixed with 65 ml of tap water per bottle. Inoculation of the barley was performed with uniform 5 mm agar discs of *Fusarium oxysporium*, which had been grown on a PDA medium with 3.5% salt for a period of 4 days. The bottles were incubated at 28 °C for two weeks, allowing ample growth of the fungal isolates^18^.

#### Pots preparation and inoculation

Fertile soil was sourced from the surface layer of the experimental farm at the Faculty of Agriculture, Menoufia University, and was sterilized using a 5% formalin solution. The preparation of pots and inoculation was executed as follows: The formalin-disinfected clay pots (30 cm in diameter) were filled with a mixture of sterilized soil and compost (at a 1:3 ratio) to a weight of 3 kg per pot. The potted soil was artificially infested with the inoculum at 3% (w/w) and was watered twice a week with saline water (1.5%) for seven days prior to planting. Control pots containing soil and compost (1:3) without inoculation were also prepared^18^.

Tomato seedlings of cultivar 023, which had been grown for 35 days in seed boxes filled with a peat-moss vermiculite mixture (1:1 w/w), were uprooted and transplanted into the pots at a rate of 2 seedlings per pot. Each treatment included three replicates. Immediately after transplanting, the pots were irrigated and subsequently, each seedling received 20 ml of saline water (1.5%) daily.

The treatments implemented were as follows: Treatment S = Tomato pots not treated with QSLA1isolate, and not infected with *Fusarium oxysporium subsp. lycopersici*; Treatment HS = Tomato seedlings treated with QSLA1 isolate (10^–2^) and not infected with *Fusarium oxysporium subsp. lycopersici*; Treatment FS = Tomato seedlings infected with 3% (w/w) of *Fusarium oxysporium subsp. Lycopersici* and not treated with QSLA1isolate; Treatment HFS = Tomato seedlings infected with 3% (w/w) of Fusarium oxysporium subsp. lycopersici and inoculated with QSLA1 (10^–2^). All treatments were irrigated with salt water (1.5%).

The wilt disease incidence percentage (WDI%) and severity percentage (WDS%) were determined and calculated after 28 days of transplanting, based on a 0–4 scale^18^, where: 0 = No infection, 1 = Slight infection (approximately 25% of the total), characterized by one or two yellowed leaves, 2 = Moderate infection (two or three yellowed leaves, 50% wilted), 3 = Extensive infection (all leaves yellowed, 75% wilted, growth inhibited), and 4 = Complete infection (the entire plant yellowed, 100% wilted, leading to plant death). Disease severity was calculated accordingly.

$$\% {\text{ Disease severity }} = \, \left[ {\Sigma \, \left( {{\text{a }} \times {\text{ b}}} \right) \, /{\text{ N }} \times {\text{ K}}} \right] \, \times { 1}00$$

where (a) represents the number of infected plants in each category, (b) is the numerical value of that category, (N) is the total number of examined plants, and (K) signifies the highest degree of infection category. We recorded disease incidence for each individual treatment using the appropriate formula.

To evaluate the effectiveness of QSLA1 in controlling *Fusarium* wilt under salinity stress, root length, shoot height, and shoot infected length were measured with precision. The shoot infected length was defined as the length of the browned area at the bottom of the shoot, conclusively indicating the presence of the pathogen^52^.

### Statistical analysis

The data underwent rigorous statistical analysis using analysis of variance (ANOVA). Differences between means were firmly evaluated using a high-range statistical domain with Tukey’s post hoc analysis, enabling us to distinguish between homogeneous and heterogeneous groups across various variables. We established significance for multiple comparisons of means at a probability level of (*p* = 0.05). The results are presented as average means ± standard deviations (SD) from triplicate measurements, ensuring robust and reliable findings.

## Results and discussion

### Isolation and purification of halophilic bacteria from solar saltern water

#### Bacterial isolates

Bacterial isolates, QSLA1 and QSLA3, were successfully obtained from the second pond with an 8.2% salt concentration, while the QSLA2 strain was isolated from the third pond, exhibiting a salinity of 17.2%. These isolates were effectively cultured on nutrient agar (NA) culture medium prepared with saline water sourced directly from the pond samples, rather than distilled water.

#### Cultural, morphological, and biochemical characterization of the isolates

Cultural, morphological, and biochemical characterizations, which play a critical role in the partial identification of microorganisms^54^ were comprehensively conducted. It was confirmed that all three isolates were gram-negative, motile, catalase-positive, and rod-shaped. Notably, they were not spore forming bacteria, as documented in Tables 2 and 3. These findings reinforce our understanding of the isolates’ properties and their potential applications.

**Table 2:** Colony morphology of isolates on nutrient agar plates (12.5% NaCl).

Tested Characters	Colony shape	Colony edge	Opacity	Elevation	Size	Pigmentation	Appearance
Isolates							
QSLA1	Round	Lobate	Translucent	Flat	Pin point	Creamy	Shinny
QSLA2	Round	Lobate	Opaque	Raised	Small	White	Shinny
QSLA3	Irregular	Ragged	Translucent	Flat	Small	Creamy	Shinny

**Table 3:** Morphological and biochemical characterization of the isolates.

Tested Characters	Shape	Concentration of the isolation pond(% NaCl)	Gram stain	Catalase test	Motility	Spore forming
Isolates						
QSLA1	Rod shape	8.2	G^−^	+	+	−
QSLA2	Rod shape	17.2	G^−^	+	+	−
QSLA3	Rod shape	8.2	G^−^	+	+	−

#### Salinity tolerance of the isolates

The salinity tolerance tests clearly demonstrated that none of the isolates could grow without salt (0% NaCl). In contrast, all isolates thrived on NA culture medium containing 7% and 12.5% NaCl. Notably, QSLA1 and QSLA2 exhibited remarkable growth on NA culture medium with 20% NaCl, with QSLA2 even thriving in medium with 22% NaCl (see Table 4).

**Table 4:** Salinity tolerance assay of the isolates (NaCl %).

Isolates	NaCl concentrations (%)				
	Zero	7	12.5	20	22
QSLA1	−	+	+	+	−
QSLA2	−	+	+	+	+
QSLA3	−	+	+	−	−

Moderate halophiles typically prosper in environments with 0.5 to 2.5 M NaCl (approximately 3% to 15% NaCl), while extreme halophiles flourish in conditions with 2.5 to 5.2 M (saturated) NaCl (15% to 30% NaCl) and cannot survive without salt. Thus, the inability of these isolates to grow in the absence of NaCl decisively confirms their classification as halophilic bacteria. Among them, QSLA1 and QSLA3 are confidently identified as moderately halophilic, while QSLA2 is undoubtedly recognized as an extreme halophilic bacterium^36^.

Halophilic bacterial strains were successfully isolated from three distinct basins of Lake Meyghan, each characterized by varying salinity levels: the green brine with approximately 50 g/L salinity, the red brine with around 180 g/L salinity, and the white brine with about 300 g/L salinity^39^. In a separate study, moderately halophilic bacteria were isolated from hypersaline environments, specifically solar salterns and salt lakes in Alexandria, Egypt. Notably, 85% of their isolates were Gram-negative, while the remaining 15% were Gram-positive^17^. Forty-six halo-bacterial isolates were identified from soil and water samples at Sambhar Lake. Remarkably, all isolates demonstrated tolerance to 10% NaCl, with forty-four cultures exhibiting resilience to 15% NaCl, and three out of ten selected cultures tolerating as much as 25% salt^40^.

#### Molecular identification of the isolates via 16S rDNA

In addition to performing cultural, morphological, and biochemical characterization, the 16S rDNA genes of the isolates were sequenced and analyzed for molecular identification. The resultant sequences were meticulously compared against those in the NCBI BLAST database. The analysis revealed that isolate QSLA1 is closely related to the *Halomonas* sp. strain RS-17, with a strong similarity of 91.26%. Isolate QSLA2 was identified as *Halomonas* sp. strain LR2-3, showing 96.6% similarity, while QSLA3 was confirmed as *Halomonas* sp. GQ30, exhibiting an impressive 97.33% similarity.

#### Evolutionary relationships of taxa

**Fig. 1:**
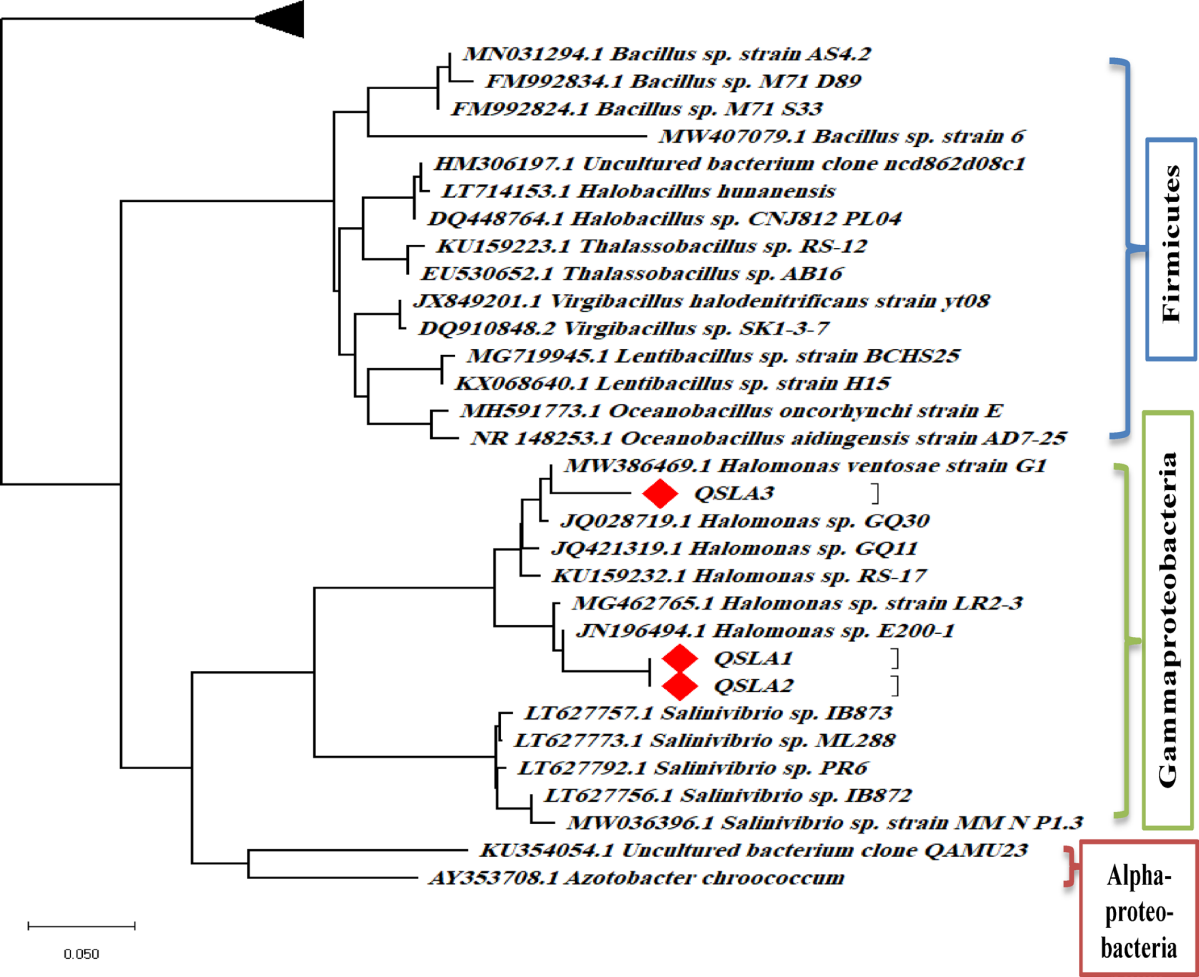
Phylogenetic tree constructed using neighbor-joining analysis based on 16S rDNA sequences from the isolates and closely related sequences deposited in GenBank.

Using the Clustal W program, the sequences were aligned and the MEGA X program was employed to construct a phylogenetic tree. Results clearly demonstrate that these isolates belong to the class Gammaproteobacteria, as depicted in Fig. 1. This robust phylogenetic affiliation underscores the significance of our research in understanding halophilic microbial diversity.

The three isolates have been deposited in the NCBI GenBank under the accession numbers listed in Table 5.

**Table 5:** Accession numbers of the sequences of submitted isolates deposited in NCBI GenBank.

Isolates	Organisms	Strains in genebank	Accession numbers
QSLA1	*Halomonas*	*Halomonas* sp. strain QSLA1	OP442496
QSLA2	*Halomonas*	*Halomonas* sp. strain QSLA2	OP442497
QSLA3	*Halomonas*	*Halomonas* sp. strain QSLA3	OP442498

361 halo bacterial strains were isolated from three distinct basins of Lake Meyghan, each exhibiting varying salinities. These strains are classified into several key classes, including Gammaproteobacteria, Alphaproteobacteria, Bacteroidetes, and Firmicutes, representing a diverse range of genera^39^.

74 halophilic bacteria were identified from the saline ecosystems of Algeria’s Sebkha and Chott lakes, which are located in arid and semi-arid eco climate zones. Notably, 16 of these isolates were closely related to the genus *Halomonas*^35^. Moderate halophilic strains were isolated from solar salterns, and their phylogenetic analysis confirmed affiliations with five notable genera: *Bacillus, Halobacillus, Planococcus, Salinicoccus,* and *Halomonas*^8^.

Taxonomic analyses of moderately halophilic bacteria isolated from hypersaline habitats, specifically solar salterns and salt lakes in Alexandria, Egypt, revealed that a remarkable 85% of the isolates belonged to the γ-Proteobacteria. Five genera were precisely identified: *Pseudoalteromonas, Flavobacterium, Chromohalobacter, Halomonas,* and *Salegentibacter*^17^.

Romania’s salt lakes, which have salinities exceeding 70 g/L, are home to bacteria from three predominant phyla: Firmicutes, Proteobacteria, and Actinobacteria, with *Halomonas* standing out as the most representative genus within the Proteobacteria phylum^46^.

Importantly, in vitro assessments of plant growth-promoting traits underscore the significant potential of halophilic and halotolerant bacteria to enhance plant growth. This approach is essential for the development of effective bio-inoculants for saline soils^40^. All isolates demonstrated robust plant growth-promoting activities, such as phosphorus solubilization, production of indole-3-acetic acid (IAA), and nitrogen fixation (Table 6).

**Table 6:** Plant growth promoting ability results of the isolates.

Isolates	Nitrogen fixation (Ashby medium)	IAA production	Phosphate solubilization
QSLA1	−	+	−
QSLA2	+	+	+
QSLA3	−	+	−

#### Nitrogen fixation and phosphate solubilization assay

**Fig. 2:**
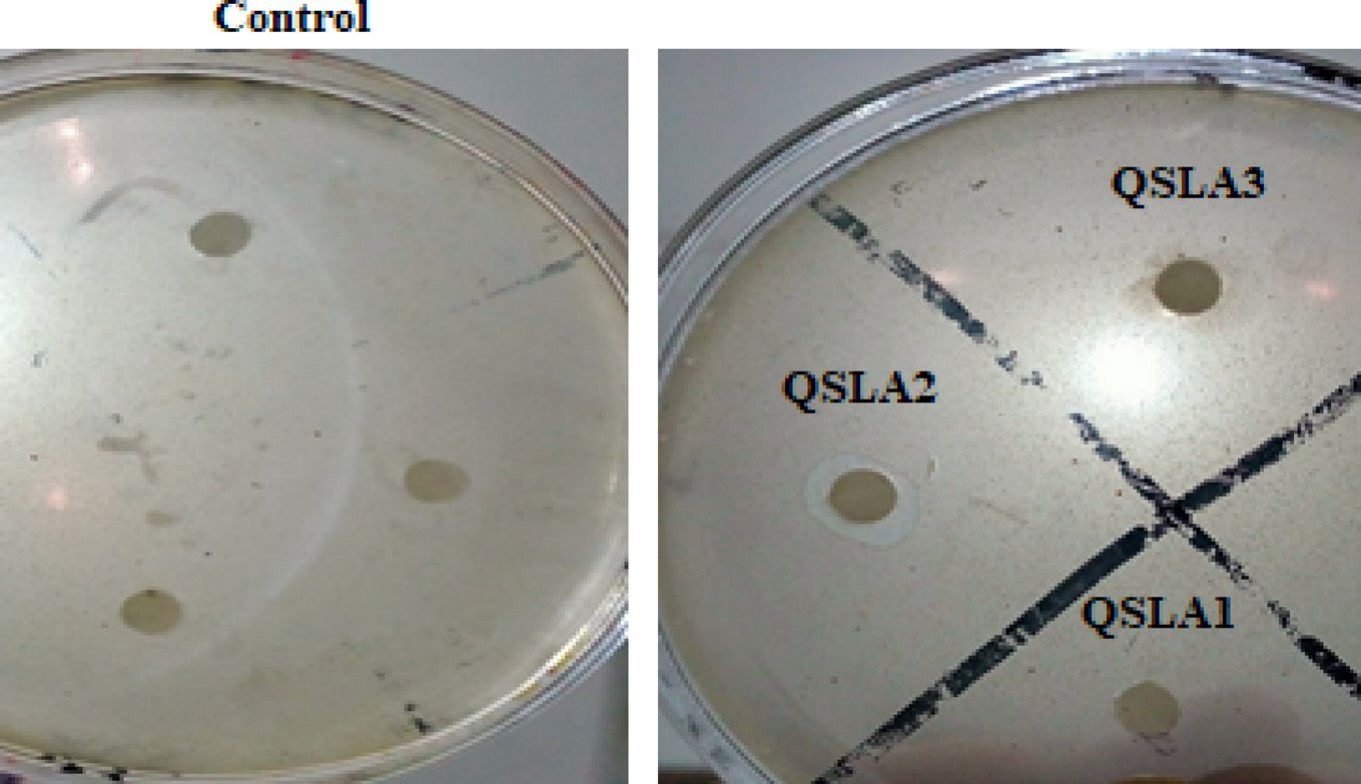
PVK agar plates showing distinctly clear zones around the positive bacterial isolates with 12.5% NaCl.

The results clearly demonstrate that the QSLA1 and QSLA3 isolates do not have the capability to fix nitrogen, as they failed to grow on Ashby N-free culture medium. Furthermore, they do not solubilize phosphate, as indicated by the lack of clear zones around their colonies on Pikovskaya (PVK) agar plates (Fig. 2). In stark contrast, the QSLA2 isolate has proven to be effective in both solubilizing phosphate (Fig. 2) and fixing nitrogen.

### Indole acetic acid (IAA) production assay

**Fig. 3:**
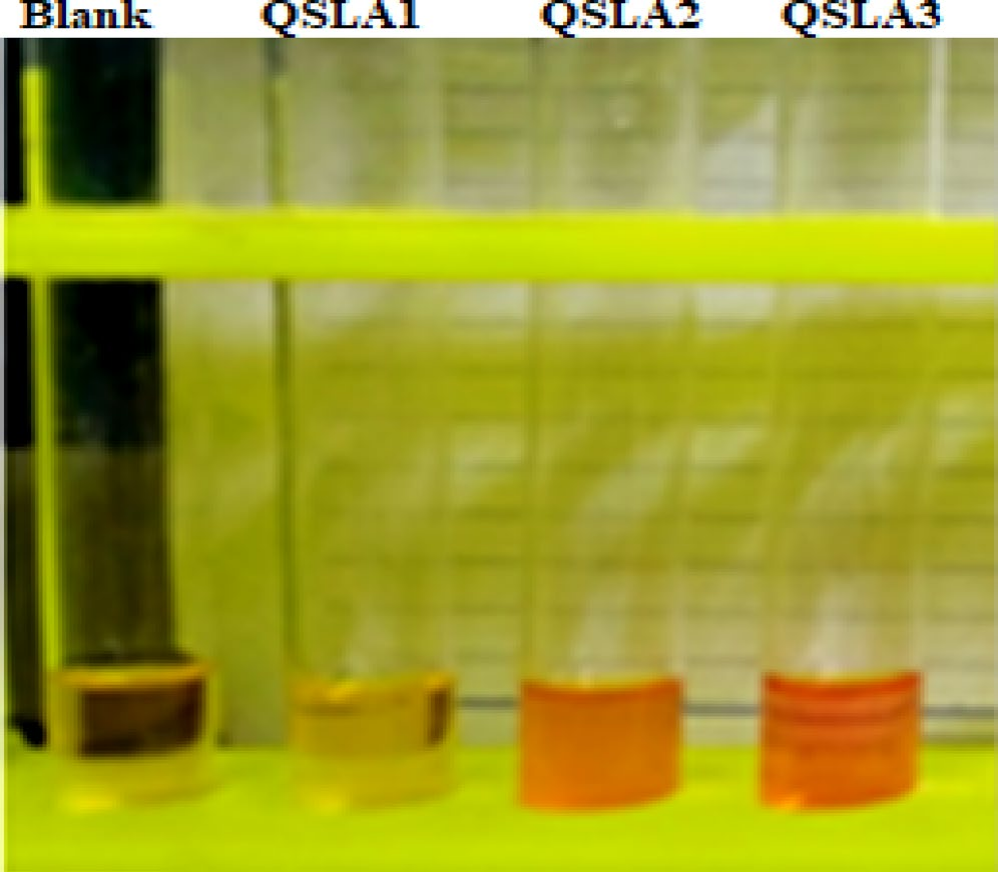
demonstrates the vibrant color development resulting from the Indole-Salkowski reagent reaction in nutrient broth medium enriched with 1% L-tryptophan.

**Fig. 4:**
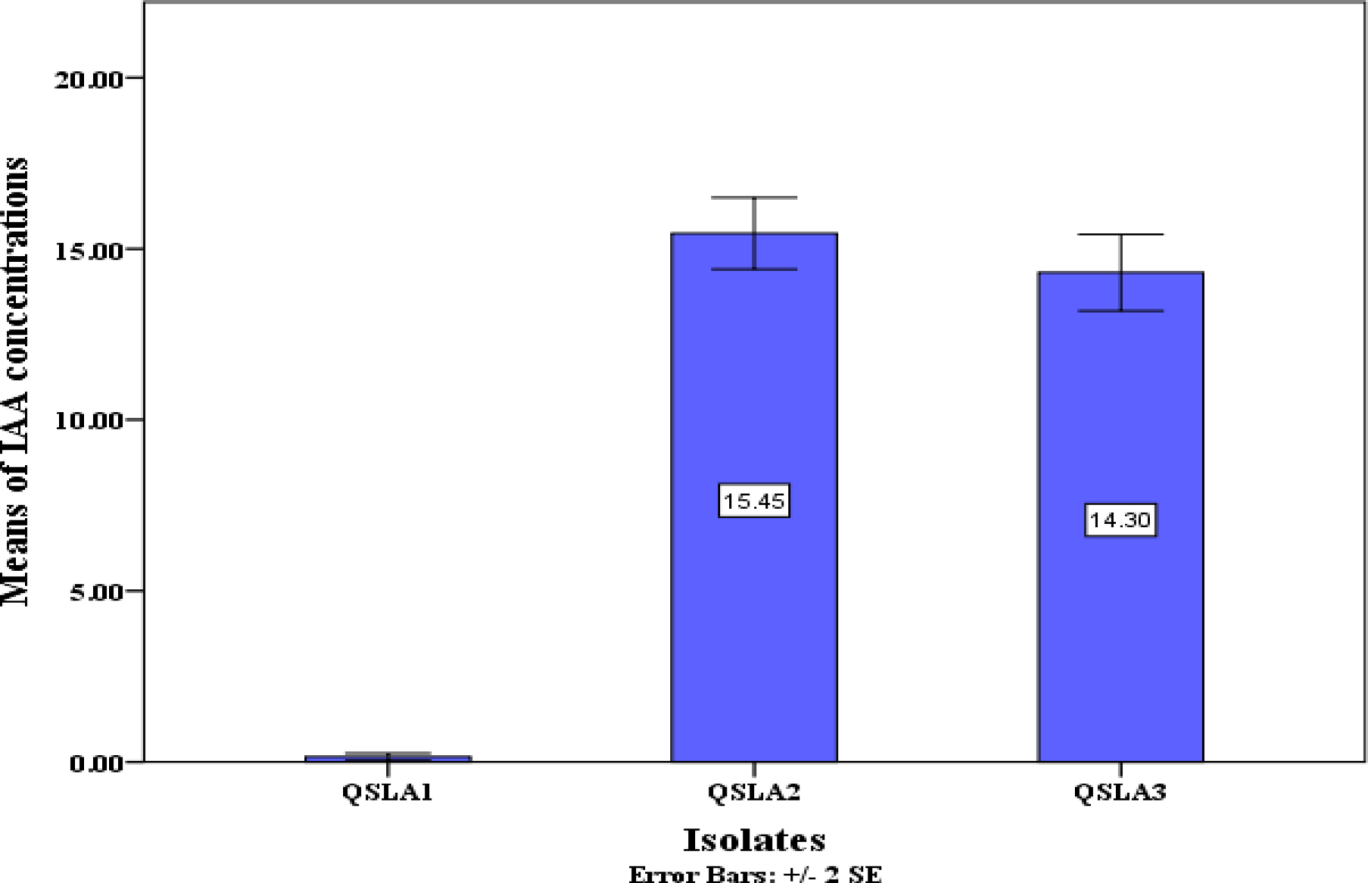
Concentrations of IAA produced in NB medium supplemented with 1% l-tryptophan.

All isolates tested demonstrated the ability to produce Indole acetic acid (IAA) in NA culture medium enriched with L-tryptophan (see Table 7 and Fig. 3). Notably, the QSLA1 isolate achieved the highest IAA production at 15.45 µg/ml, followed closely by QSLA3 at 14.30 µg/ml (refer to Table 7). The differences in IAA production between the QSLA1 isolate and the other two isolates were statistically significant, while the QSLA2 and QSLA3 isolates showed no significant differences in their production levels (see Table 7 and Fig. 4).

**Table 7:** IAA concentration produced by the isolates (means + standard deviation).

Isolates	IAA concentration (µg/ml) ± S.D
QSLA1	0.15 ± 0.09^a^
QSLA2	15.45 ± 0.91^b^
QSLA3	14.30 ± 0.97^b^

Values are means ± standard deviation of three replicates.

b = higher value, a = lower value.

120 salt-tolerant bacterial strains were successfully isolated from various locations along Portugal’s coastline. Among these, *Halomonas titanicae* was particularly noteworthy for its capacity to fix nitrogen, solubilize phosphate, and produce IAA at a concentration 12.22 µg/ml^15^. Furthermore, a salt-tolerant strain, *Halomonas* sp. that exhibited impressive plant growth-promoting traits including phosphate solubilization and nitrogen fixation in high-salinity habitats in India was isolated^57^. The exceptional growth-promoting potential of the moderate halophile *Halomonas* sp. MAN5 was underscored, and it was found to have the potential to produce 95.3 µg/ml of IAA and solubilize 53 parts per million (ppm) of phosphates in the presence of 15% NaCl^11^.

The variation in IAA concentrations among the different bacterial isolates can be attributed to their distinct abilities to utilize tryptophan or to the diverse pathways involved in IAA biosynthesis, including Indole-3-Pyruvic Acid, Tryptamine, and Indole-3-Acetonitrile^26^.

### In vitro antagonistic activity of the isolates against plant pathogens

**Fig. 5:**
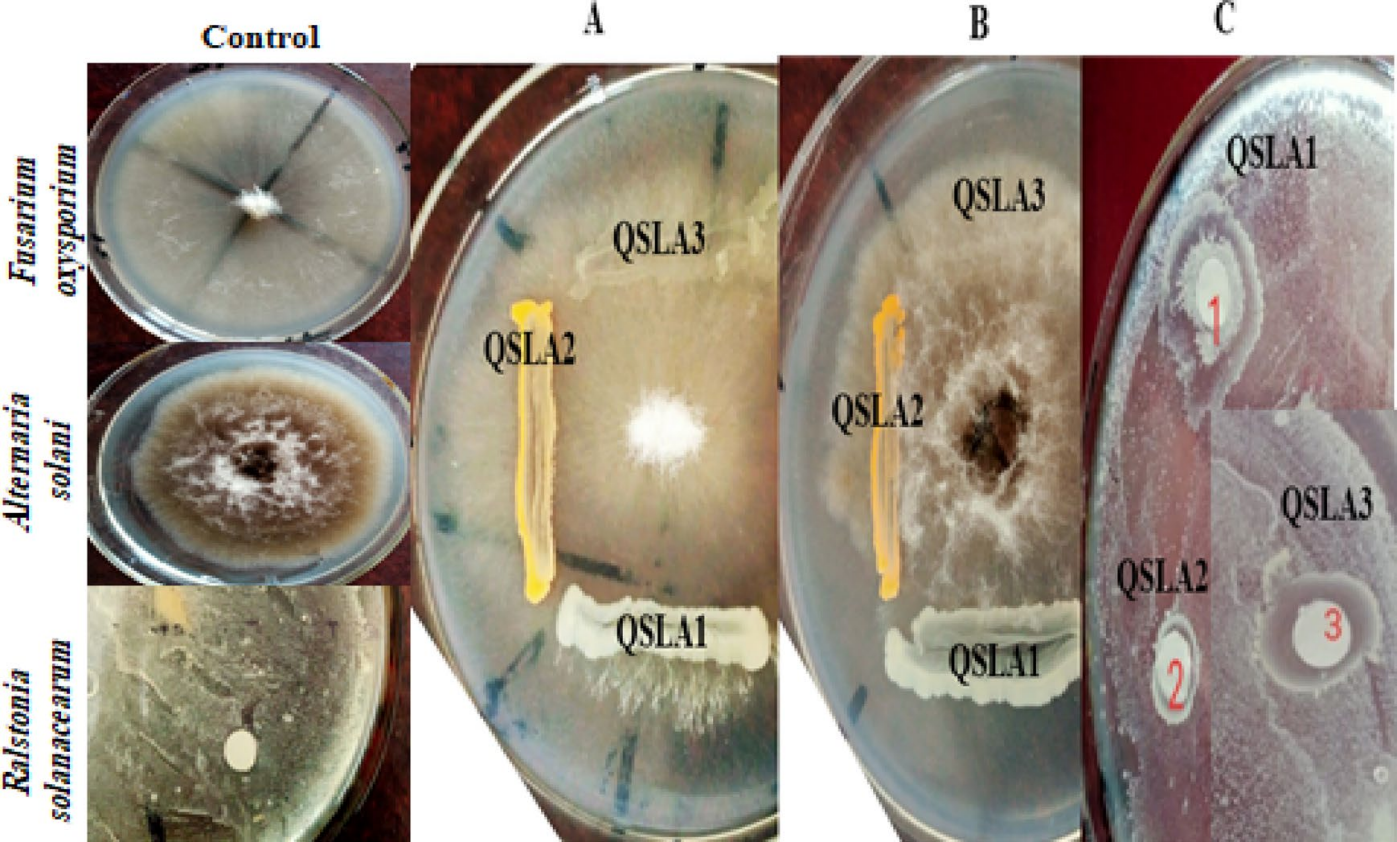
the antagonistic effects of the isolates against *Fusarium oxysporium* (**A**), *Alternaria solani* (**B**), and *Ralstonia solanacearum* (**C**) were compellingly demonstrated at a 12.5% NaCl concentration.

Isolate QSLA1 exhibited notable antifungal activity against *Fusarium oxysporium f.sp. Lycopersici* and *Alternaria solani*, effectively limiting mycelial growth in a co-cultivation plate assay. Additionally, it demonstrated significant antibacterial activity against *Ralstonia solanacearum* using the disc diffusion method at a 12.5% salt concentration. In contrast, isolates QSLA2 and QSLA3 displayed antibacterial activity exclusively against *Ralstonia solanacearum* (see Table 8 and Fig. 5). Consequently, isolate QSLA1 has been selected for further studies due to its impressive antagonistic activity against multiple pathogens.

**Table 8:** Results of antagonistic activity of the isolates against plant pathogens in vitro.

Isolate	*Fusarium oxysporium*	*Alternaria solani*	*Ralstonia solanacearum*
QSLA1	+	+	+
QSLA2	−	−	+
QSLA3	−	−	+

### Determination of bioactive chemical constituents produced by QSLA1 isolate using GC–MS analysis

**Fig. 6:**
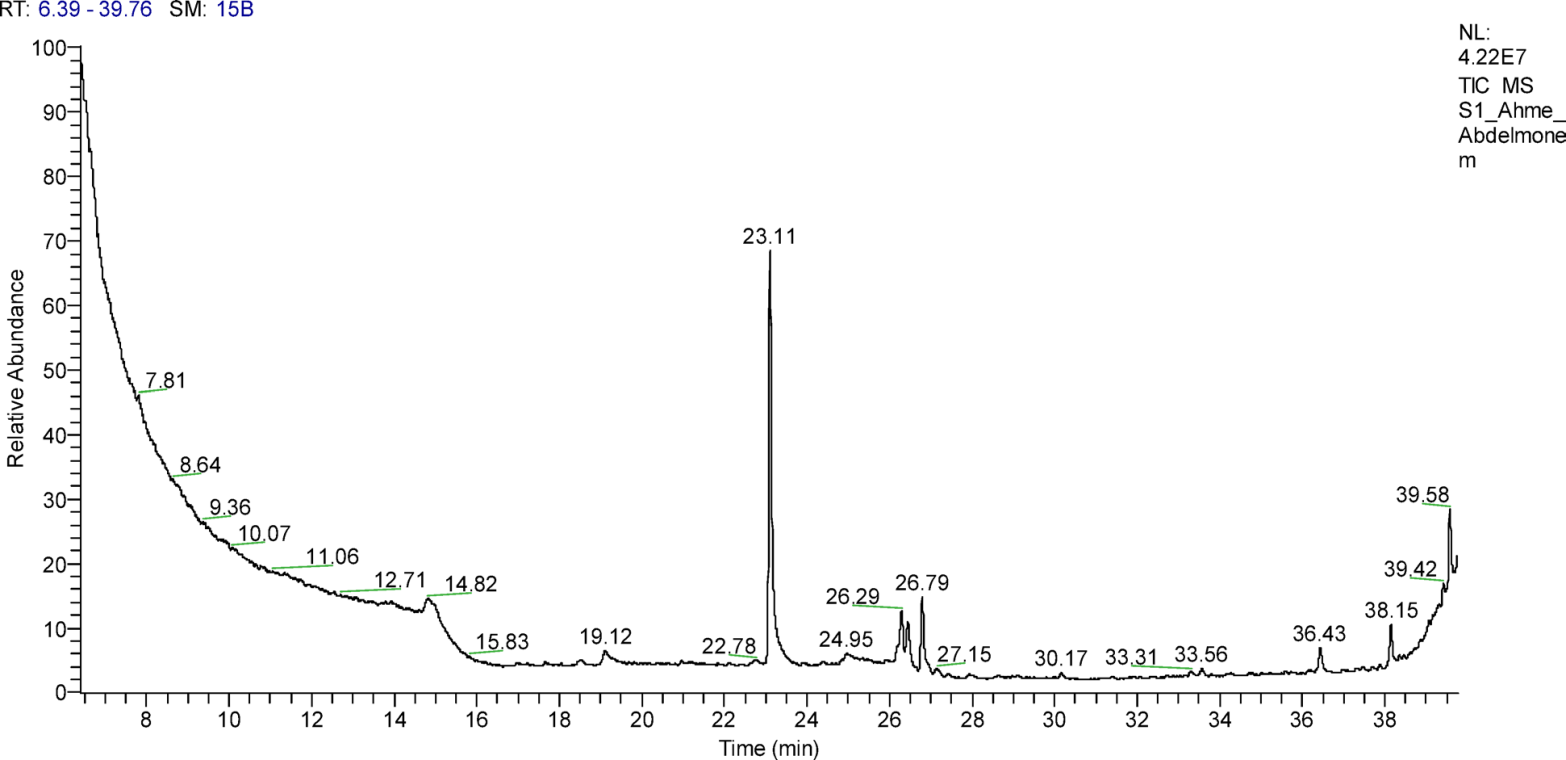
The GC–MS chromatogram from the supernatant of the QSLA1 isolate reveals a rich array of bioactive compounds.

As its antagonistic activity against all used phyto-pathogens, the bioactive chemical constituents produced by the QSLA1 isolate were successfully identified through comprehensive GC–MS analysis. Insights from unpublished bioinformatics data prompted a decisive investigation into the bioactive compounds released by halophilic isolates. As a result, the extract from QSLA1 underwent thorough GC–MS analysis (see Fig. 6), revealing its metabolic profile and identifying the specific chemical compounds present (refer to Table 9).

**Table 9:** The chemical compounds detected in the culture supernatant of QSLA1 after centrifugation, as revealed by the GC–MS analysis, clearly underscore the remarkable bioactive potential of this isolate.

No	Compound name	Rt (Min)	Area %	Molecular formula	Molecular weight	Function (PubMed)
1	Desulphosinigrin	13.70	0.23	C_10_H_17_NO_6_S	279	Antibacterial
2	Decanoic acid, ethyl ester	15.29	0.04	C_12_H_24_O2	200	Antibcterial
3	Undeca-2,4,6,8,10-pentaenal, 11-(2-Furyl)-, oxime	16.35	0.10	C_12_H_26_O_5_S	282	Antibacterial/Antifungal
4	Hexadecanoic acid, methyl ester	23.11	33.22	C_17_H_34_O_2_	270	Antimicrobial
5	Strychane, 1-acetyl-20à-hydroxy-16-methylene	23.97	0.11	C_21_H_26_N_2_O_2_	338	Antimicrobial
6	Curan-17-oic acid, 19,20-dihydroxy-, methyl ester, (19S)	24.94	1.39	C_20_H_26_N_2_O_4_	358	Antimicrobial
7	Cyclopropaneoctanoic acid, 2-[[2-[(2-ethyl cyclopropyl) methyl] cyclopropyl]methyl]-, methyl ester	26.19	0.071	C_22_H_38_O_2_	334	Antimicrobial
8	Palmitic acid	30.16	0.59	C_16_H_32_O_2_	256	Antibacterial
9	cis-Vaccenic acid	30.63	0.10	C_18_H_34_O_2_	282	Antimicrobial
10	Ethanimidothioic Acid, 2-(Dimethylamino)-N-[[(Methylamino)Carbonyl] Oxy]-2-Oxo-, Methyl Ester	34.26	0.27	C_7_H_13_N_3_O_3_S	219	Insecticide

Notably, the main chemical constituents identified include Hexadecanoic Acid, Methyl Ester (33.22%), 9-Octadecenoic Acid, (2-Phenyl-1,3-Dioxolan-4-Yl)Methyl Ester, Cis (6%), and d-Lyxo-d-manno-nononic-1,4-lactone (2.94%). Other significant compounds are Curan-17-oic acid, 19,20-dihydroxy-, methyl ester, (19S) (1.39%), Cyclopropanedodecanoic acid, 2-octyl-, methyl ester (1.39%), and several others, including 13,16-Octadecadiynoic Acid, Methyl Ester (1.27%) and Palmitic Acid (0.59%). The majority of these constituents are fatty acids and fatty acid derivatives, recognized for their biosurfactant properties and antimicrobial activity ^10,16^. Additionally, compounds such as Reynosin (lactone) (0.25%) and Leukotriene F4 (0.19%) showcase notable bioactive potential, contributing to antifungal, antitumor, antibacterial, and antioxidant properties^28^. The dominant presence of fatty acids in the extract underscores their therapeutic significance. Fatty acids and their derivatives were emphasized as versatile agents against various health issues, including cancer and bacterial infections^55^. Furthermore, acids like 3-hydroxydecanoic acid and decanoic acid, sourced from *Lactobacillus plantarum*, exhibit strong antibacterial properties^32^. Fatty acids have also been shown to disrupt bacterial growth by altering membrane permeability and inhibiting fatty acid synthesis^56^. Moreover, the compounds (antibiotics and biosurfactants) derived from *Halomonas meridiana* BK-AB4 playing a vital role in combating pathogens^50^.

### Controlling tomato fusarium wilt pathogen with QSLA1 (*Halomonas* sp.)

**Fig. 7:**
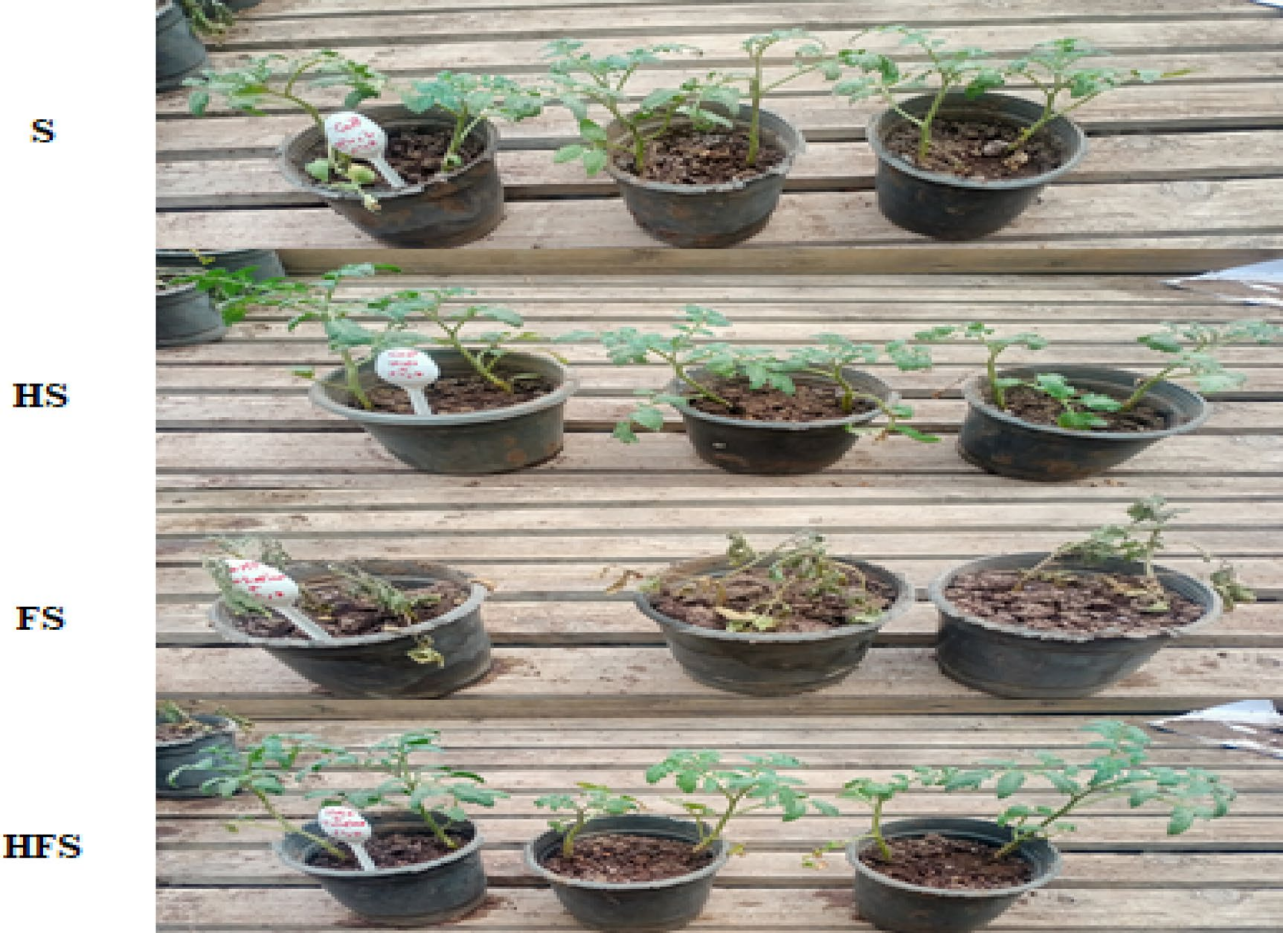
A greenhouse experiment clearly demonstrated significant differences among treatments after 28 days. Treatment S = tomato seedlings irrigated with salt water (1.5%), not treated with QSLA1and not infected with *Fusarium*. Treatment HS = tomato seedlings treated with QSLA1 isolate (10^–2^) and not infected with *Fusarium*. Treatment FS = tomato seedlings infected with 3% (w/w) of *Fusarium oxysporium sub sp. lycopersici*, and not treated with QSLA1. Treatment HFS = tomato seedlings infected with 3% (w/w) of *Fusarium oxysporium sub sp. Lycopersci* and inoculated with QSLA1 (10^–2^). All treatments irrigated with salt water (1.5%).

A pot experiment has successfully demonstrated the efficacy of QSLA1 against *Fusarium oxysporium f.sp. lycopersici*, showcasing its remarkable ability to control *Fusarium* wilt disease in tomato seedlings, particularly under saline conditions (1.5% salt water) (Fig. 7).

**Fig. 8:**
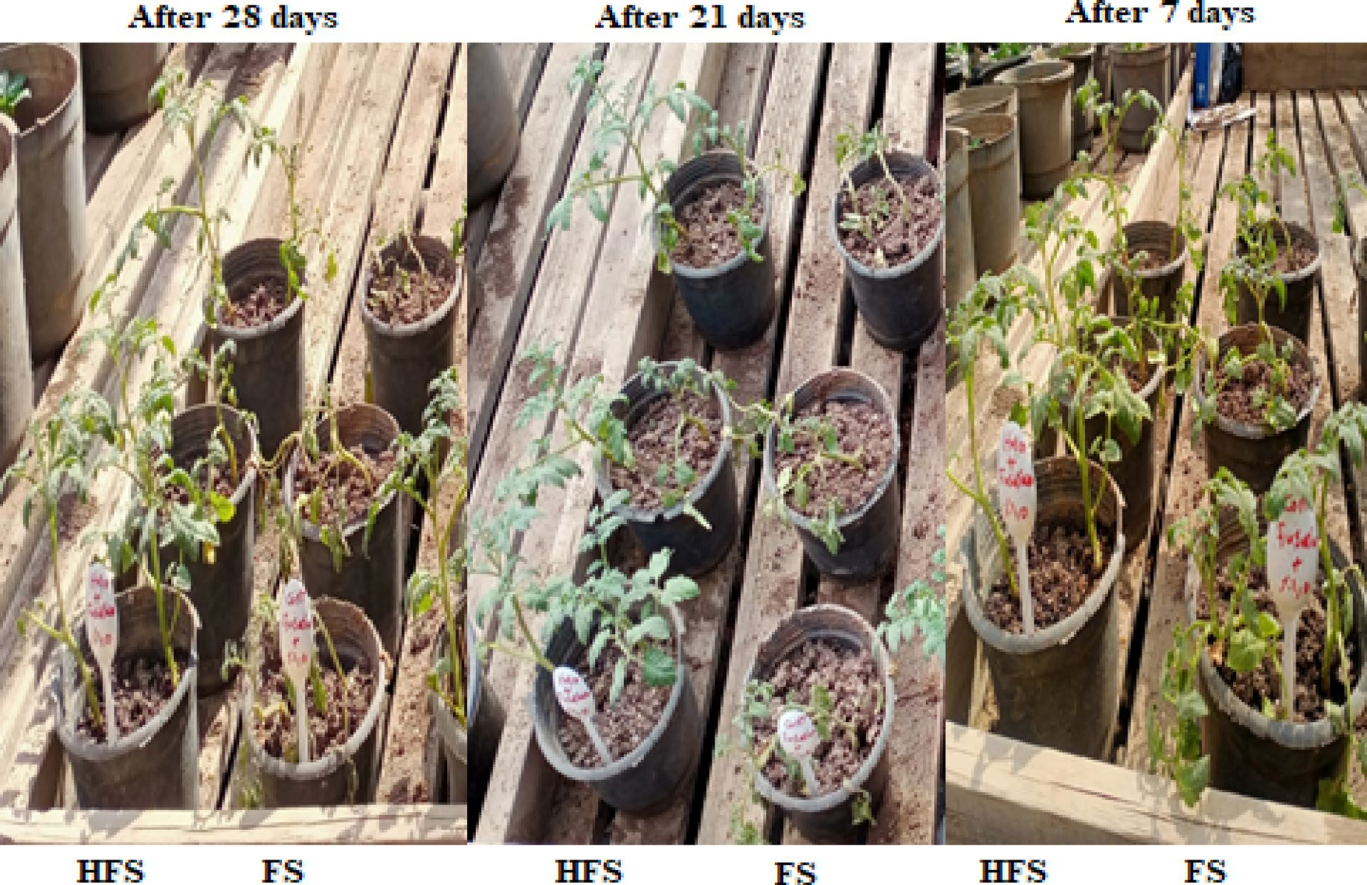
Clearly demonstrates the progression of *Fusarium* wilt symptoms throughout the experiment. Treatment S = tomato seedlings irrigated with salt water (1.5%), not treated with QSLA1and not infected with *Fusarium*. Treatment HS = tomato seedlings treated with QSLA1 isolate (10^–2^) and not infected with *Fusarium*. Treatment FS = tomato seedlings infected with 3% (w/w) of *Fusarium oxysporium sub sp. lycopersici*, and not treated with QSLA1. Treatment HFS = tomato seedlings infected with 3% (w/w) of *Fusarium oxysporium sub sp. Lycopersci* and inoculated with QSLA1 (10^–2^). All treatments irrigated with salt water (1.5%).

Throughout the 28-day experiment, notable differences in symptoms emerged between the seedlings inoculated solely with *Fusarium* (treatment FS) and those treated with both QSLA1 isolate and *Fusarium* (treatment HFS). After just 7 days post-transplanting, yellowing of the leaves was observed, often concentrated on one side of the plant, resulting from impaired lateral water translocation^9^. This symptom was evident in both treatment F and treatment HF. By the 21-day mark, seedlings treated with *Fusarium* alone (treatment FS) displayed severe symptoms, including wilting, browning of the above-ground portion, and drooping of lower leaves (Fig. 8).

**Fig. 9:**
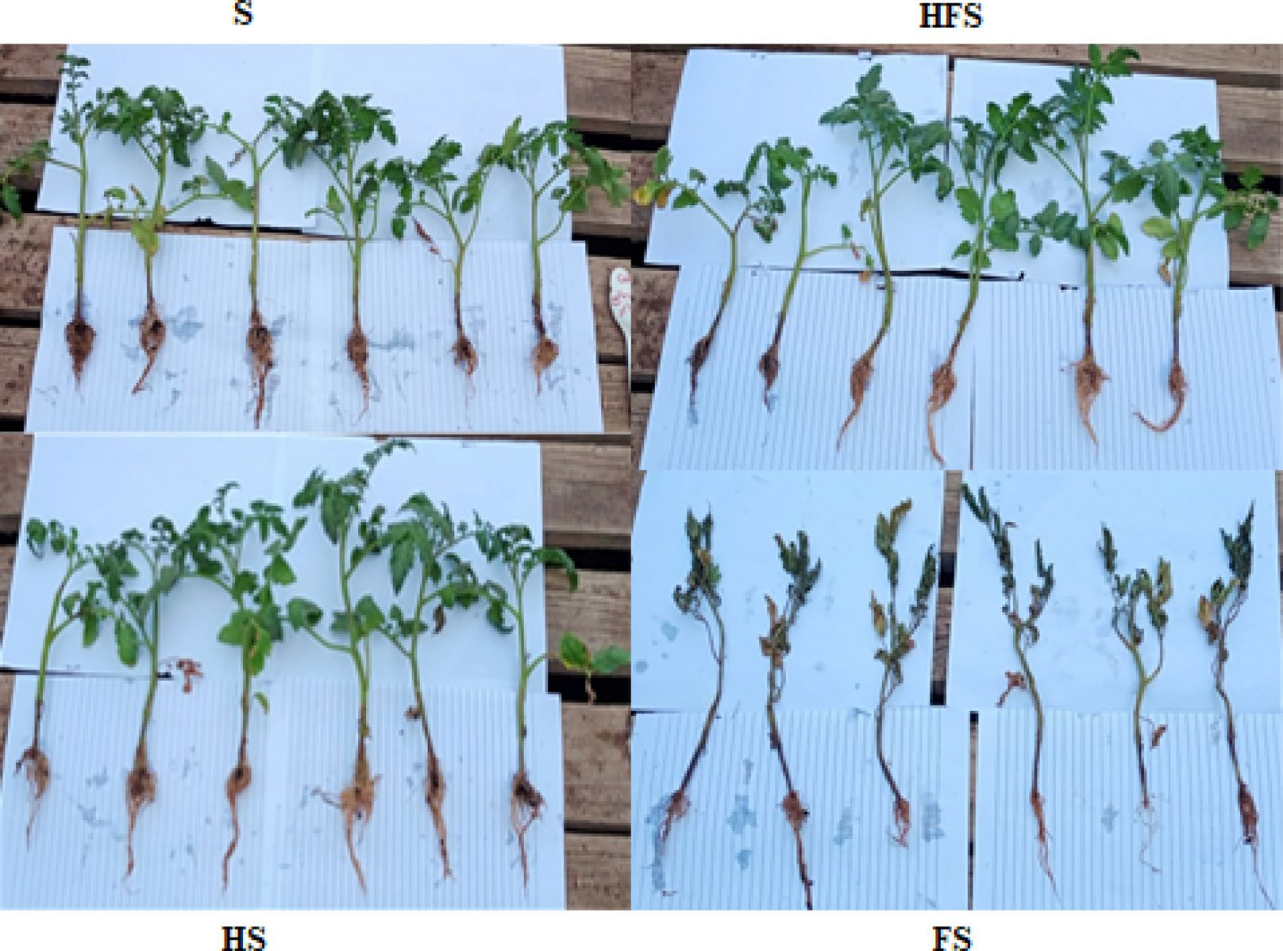
Impact of inoculation with isolate QSLA1 on tomato seedlings after 28 days. Treatment S = tomato seedlings irrigated with salt water (1.5%), not treated with QSLA1and not infected with *Fusarium*. Treatment HS = tomato seedlings treated with QSLA1 isolate (10^–2^) and not infected with *Fusarium*. Treatment FS = tomato seedlings infected with 3% (w/w) of *Fusarium oxysporium sub sp. lycopersici*, and not treated with QSLA1. Treatment HFS = tomato seedlings infected with 3% (w/w) of *Fusarium oxysporium sub sp. Lycopersci* and inoculated with QSLA1 (10^–2^). All treatments irrigated with salt water (1.5%).

The results for the other treatments reveal that seedlings inoculated with QSLA1 alone (Treatment HS) remained remarkably healthy, displaying vibrant green leaves. In stark contrast, the untreated control group (Treatment S) showed no symptoms in any of the plants, as illustrated in Fig. 9.

**Fig. 10:**
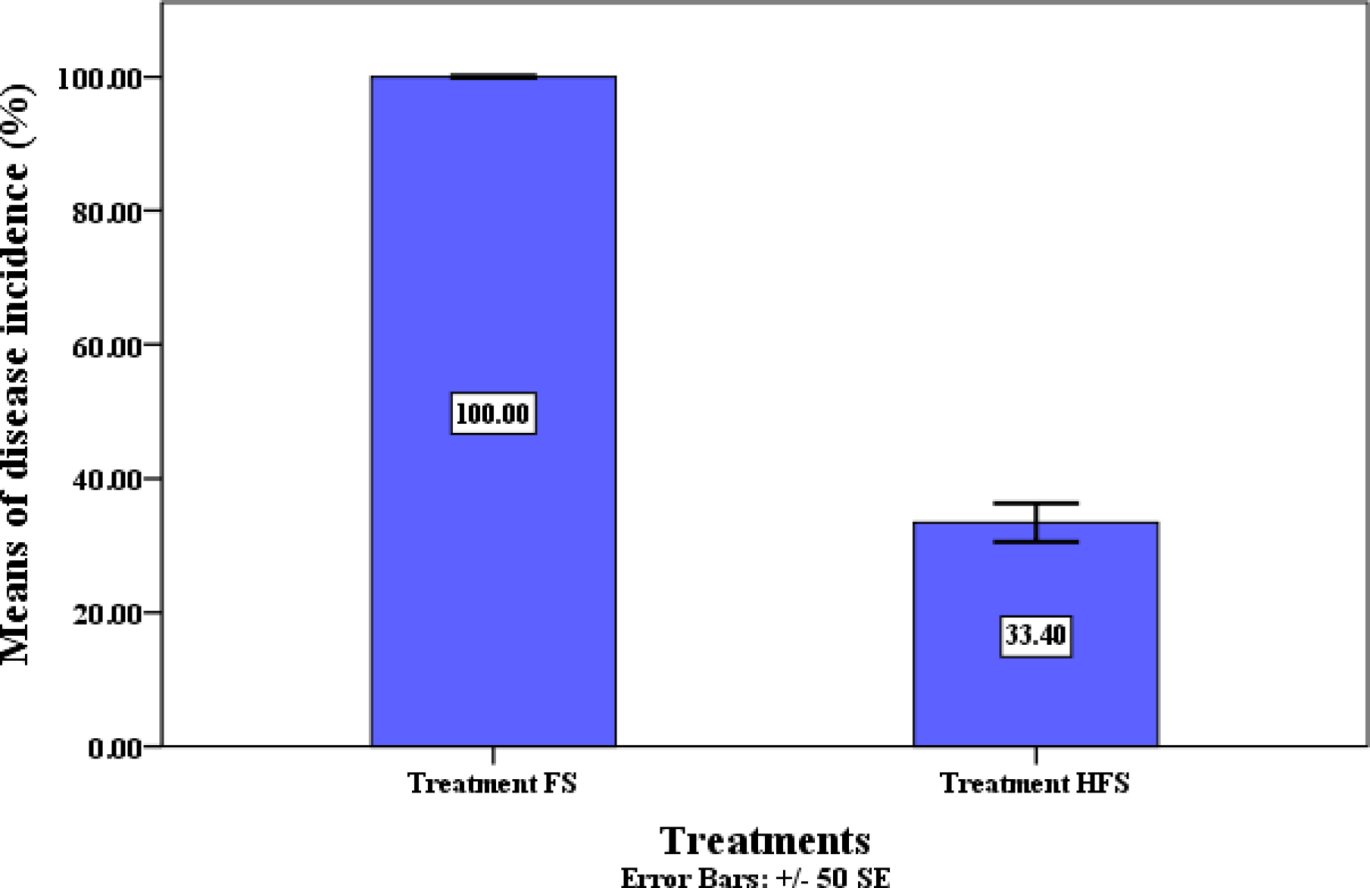
Effect of inoculation with isolate QSLA1 on disease incidence of tomato seedlings infected with *Fusarium oxysporium* sub sp. *lycopersci* after 28 days.

**Fig. 11:**
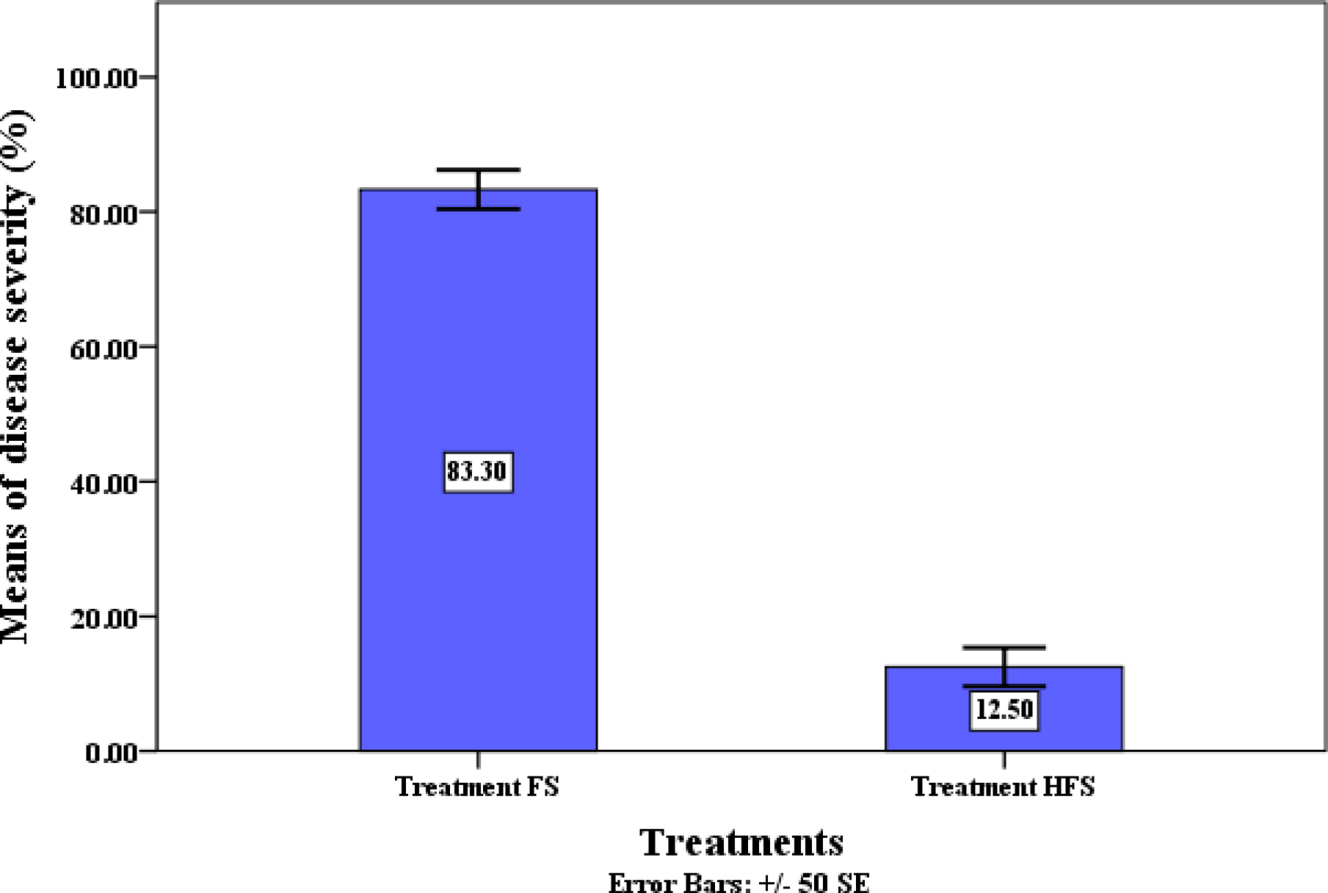
Effect of inoculation with isolate QSLA1 on disease severity percentage on tomato seedlings infected with *Fusarium oxysporium* sub sp. *lycopersici* after 28 days. Treatment FS = tomato seedlings infected with 3% (w/w) of *Fusarium oxysporium sub sp. lycopersici*, and not treated with QSLA1. Treatment HFS = tomato seedlings infected with 3% (w/w) of *Fusarium oxysporium sub sp. Lycopersci* and inoculated with QSLA1 (10^–2^). All treatments irrigated with salt water (1.5%).

Our findings clearly demonstrate that after 28 days of transplanting under greenhouse conditions, QSLA1 is highly effective in reducing the incidence of *Fusarium* wilt in tomatoes by an impressive 33.3% in seedlings treated with both QSLA1 isolate and *Fusarium* (treatment HF). In contrast, seedlings treated with *Fusarium* alone (treatment F) exhibited a 100% disease incidence (refer to Table 10 and Fig. 10). Moreover, the severity of *Fusarium* disease in seedlings treated with the QSLA1 isolate combined with *Fusarium* (treatment HF) was significantly reduced by 12.5% compared to those treated solely with *Fusarium* (treatment F), which showed an 83.3% disease severity, as confirmed by the established equation (see Table 10 and Fig. 11).

**Table 10:** The effects of inoculation with isolate QSLA1 on tomato seedlings under various treatment conditions.

Treatments	Root length (cm)	Shoot height (cm)	Shoot infection length (cm)	Disease incidence (%)	Disease severity (%)
Only fresh water	5.50 ± 0.50^a^	15.97 ± 0.25^d^	ND	ND	ND
Only salt water (1.5%) (S)	6.43 ± 0.25^b^	12.80 ± 0.20^b^	ND	ND	ND
QSLA1 only + Salt water (1.5%) (HS)	8.73 ± 0.46^d^	15.80 ± 0.20^d^	ND	ND	ND
Salt water (1.5%) + *Fusarium* only (FS)	5.07 ± 0.12^a^	11.17 ± 0.15^a^	3.63 ± 0.12^b^	100^b^	83.3^b^
QSLA1 + Salt water (1.5%) + *Fusarium* (HFS)	7.50 ± 0.36^c^	14.27 ± 0.25^c^	1.60 ± 0.36^a^	33.3^a^	12.5^a^

Values are means ± standard deviation of three replicates.

d = higher value, a = lower value, ND = not detected.

**Fig. 12:**
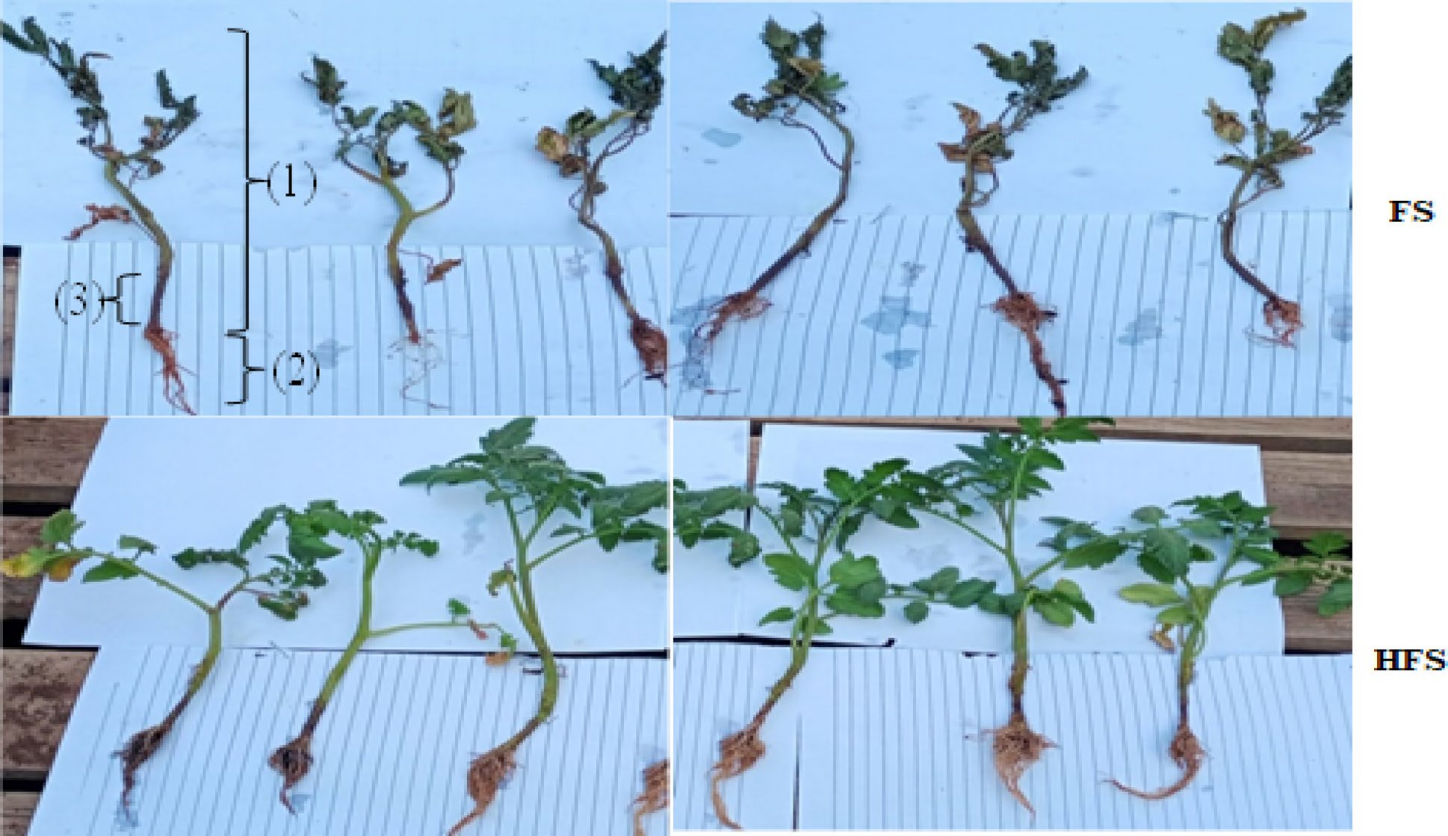
The impact of the QSLA1 isolate on shoot height (1), root length (2), and shoot infection length (3) after 28 days. Treatment FS = tomato seedlings infected with 3% (w/w) of *Fusarium oxysporium sub sp. lycopersici*, and not treated with QSLA1. Treatment HFS = tomato seedlings infected with 3% (w/w) of *Fusarium oxysporium sub sp. Lycopersci* and inoculated with QSLA1 (10^–2^). All treatments irrigated with salt water (1.5%).

**Fig. 13:**
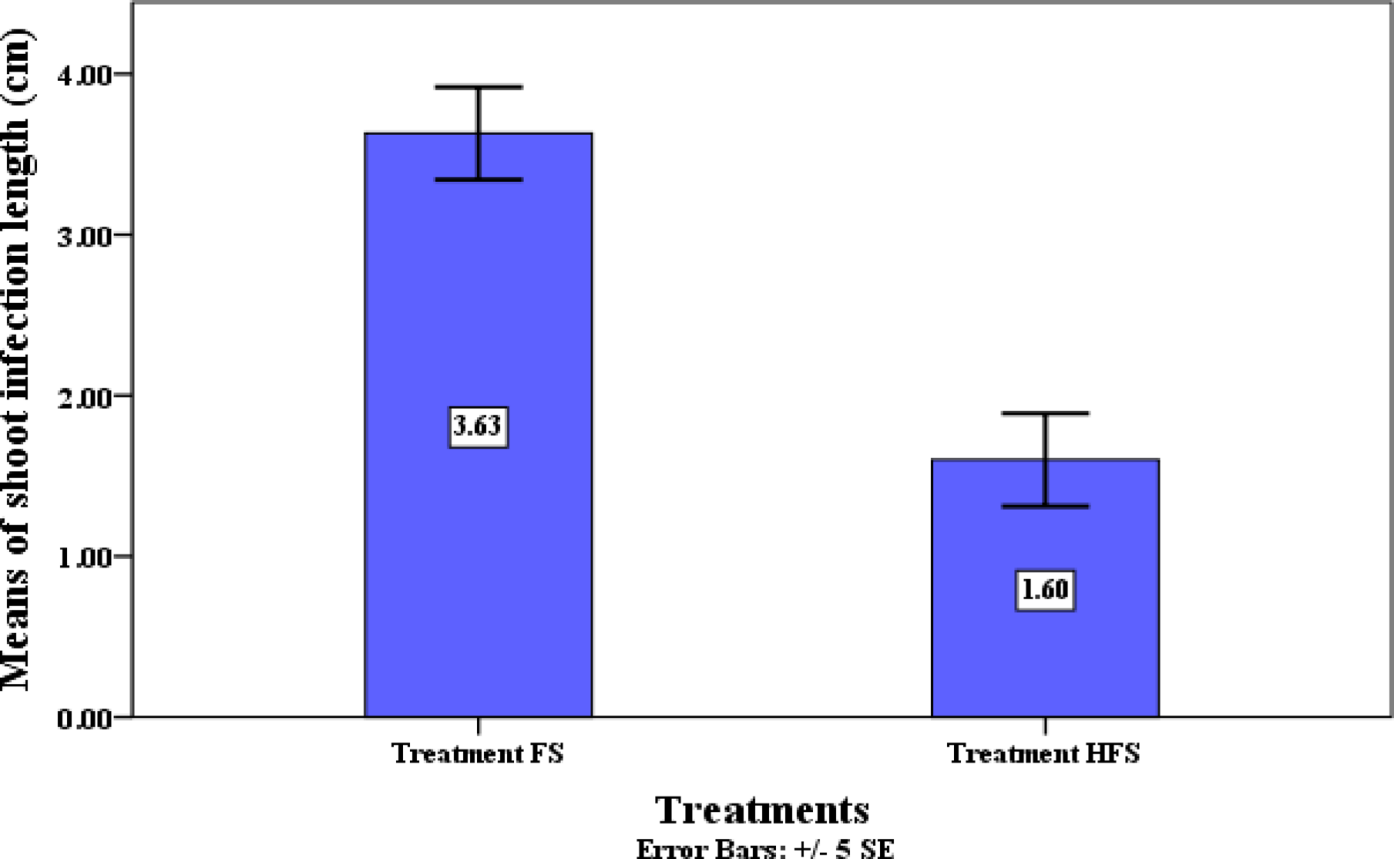
The significant impact of inoculation with isolate QSLA1 on the length of shoot infection in tomato seedlings infected with *Fusarium oxysporium subsp. lycopersici* after 28 days. Treatment FS = tomato seedlings infected with 3% (w/w) of *Fusarium oxysporium sub sp. lycopersici*, and not treated with QSLA1. Treatment HFS = tomato seedlings infected with 3% (w/w) of *Fusarium oxysporium sub sp. Lycopersci* and inoculated with QSLA1 (10^–2^). All treatments irrigated with salt water (1.5%).

Plants treated with the QSLA1 isolate in conjunction with *Fusarium* (treatment HF) and those treated with *Fusarium* alone (treatment F) exhibited a clear browning zone at the base of the seedling shoots, resulting from pathogen colonization of the tissues. This browning was quantitatively assessed as shoot infection length (see Fig. 12). The results clearly demonstrated a significant difference in shoot infection length, with tomato seedlings treated with the QSLA1 isolate and *Fusarium* (treatment HF) showing a length of 1.6 cm, compared to 3.63 cm for those treated with *Fusarium* only (treatment F) (refer to Table 10 and Fig. 13).

**Fig. 14:**
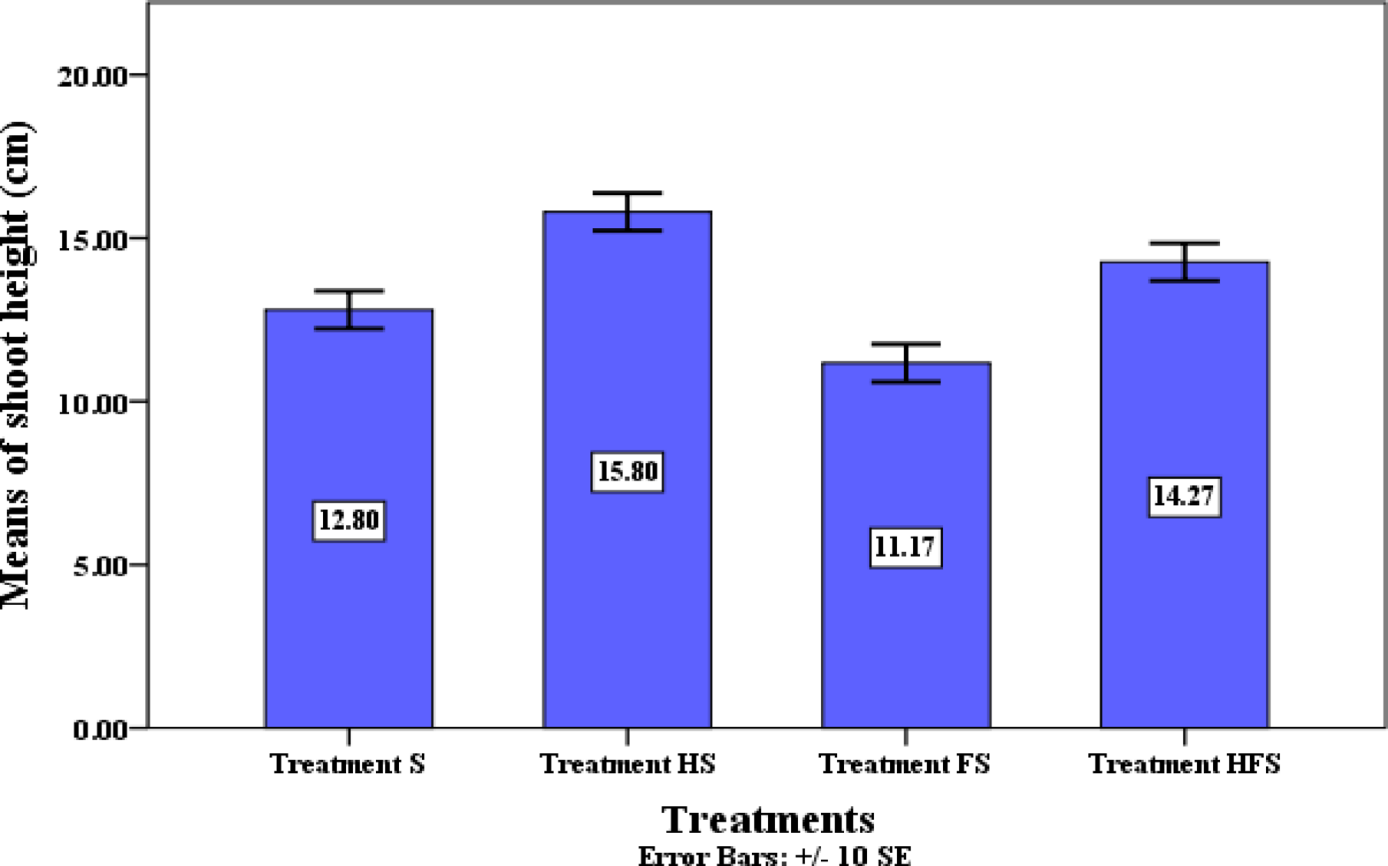
The significant impact of inoculation with isolate QSLA1 on the shoot height of tomato seedlings infected with *Fusarium oxysporium f. sp. lycopersici* after 28 days. Treatment S = tomato seedlings irrigated with salt water (1.5%), not treated with QSLA1and not infected with *Fusarium*. Treatment HS = tomato seedlings treated with QSLA1 isolate (10^–2^) and not infected with *Fusarium*. Treatment FS = tomato seedlings infected with 3% (w/w) of *Fusarium oxysporium sub sp. lycopersici*, and not treated with QSLA1. Treatment HFS = tomato seedlings infected with 3% (w/w) of *Fusarium oxysporium sub sp. Lycopersci* and inoculated with QSLA1 (10^–2^). All treatments irrigated with salt water (1.5%).

**Fig. 15:**
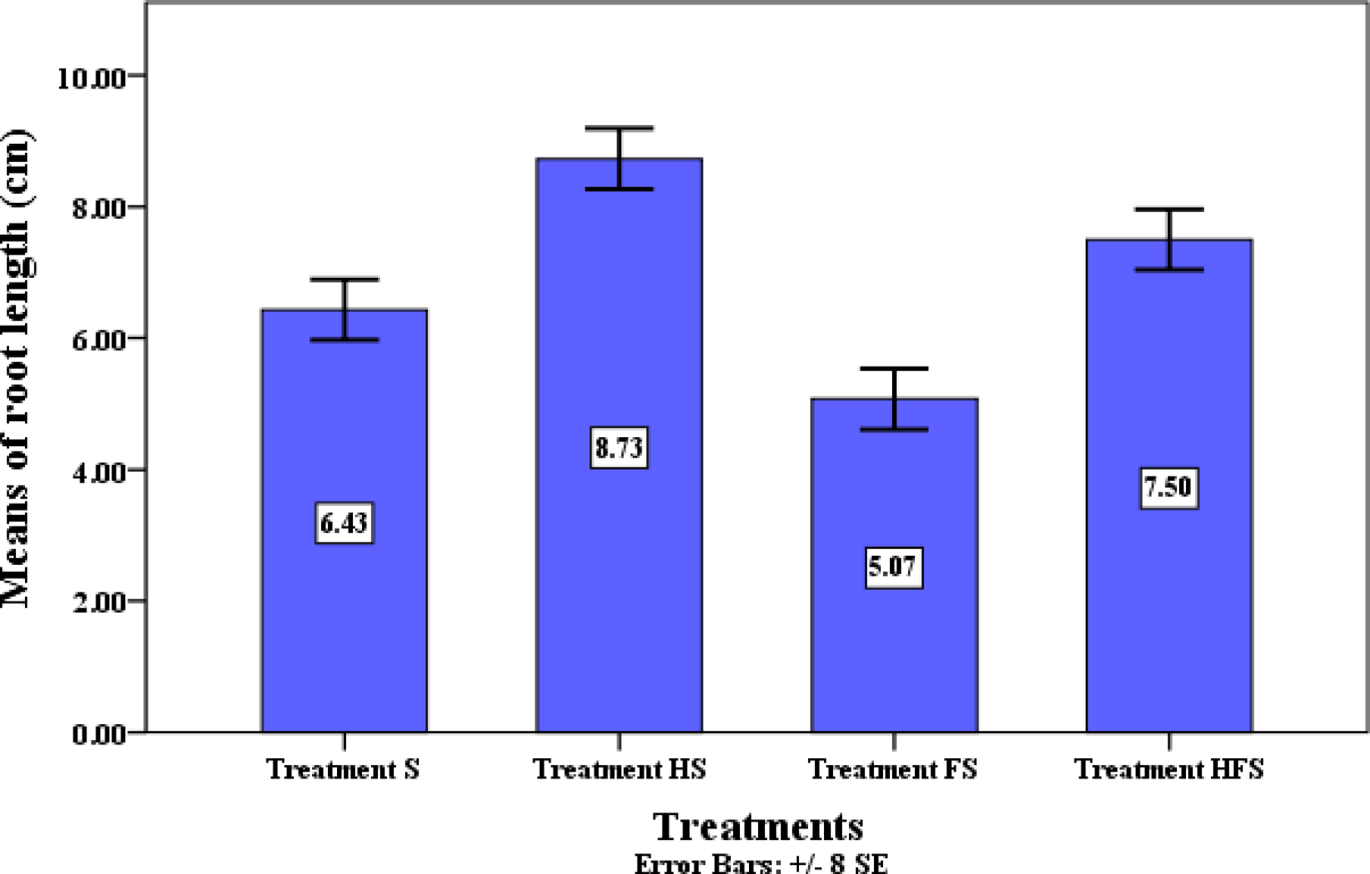
Impact of Inoculation with Isolate QSLA1 on root length of Tomato Seedlings Infected with *Fusarium oxysporium f.sp. lycopersici* after 28 Days. -Treatment S = tomato seedlings irrigated with salt water (1.5%), not treated with QSLA1and not infected with *Fusarium*. Treatment HS = tomato seedlings treated with QSLA1 isolate (10^–2^) and not infected with *Fusarium*. Treatment FS = tomato seedlings infected with 3% (w/w) of *Fusarium oxysporium sub sp. lycopersici*, and not treated with QSLA1. Treatment HFS = tomato seedlings infected with 3% (w/w) of *Fusarium oxysporium sub sp. Lycopersci* and inoculated with QSLA1 (10^–2^).—All treatments irrigated with salt water (1.5%).

Moreover, inoculation with QSLA1 significantly stimulated both shoot height and root length. The treated tomato plants achieved a shoot height of 14.3 cm and a root length of 7.5 cm, whereas seedlings treated with *Fusarium* alone (treatment F) measured 11.1 cm in height and 5.1 cm in root length (see Table 10). These differences were statistically significant and underscored the beneficial effects of QSLA1 inoculation (refer to Figs. 14 and 15).

These results underscore the significant effects of the QSLA1 isolate on plant growth and infection dynamics, also highlights the potential of isolate QSLA1 in promoting seedling growth even in the presence of this pathogen.

The infection process begins with fungal hyphae adhering to and penetrating the root surface. The mycelium invades the root cortical cells intercellularly, subsequently entering the vascular system through the xylem. This fungus exhibits a distinct infection pathway, effectively colonizing exclusively inside the xylem vessels, allowing it to rapidly infiltrate the host. Within these vessels, the fungus produces microconidia, which ascend through the sap stream. The germination of these microconidia facilitates mycelial penetration of the upper vessels. Characteristic wilt symptoms arise from vessel blockage caused by accumulating fungal hyphae, compounded by host–pathogen interactions involving the release of toxins (such as fusaric acid and lycomarasmin), gums, gels, and the formation of tyloses. Symptoms including leaf drooping, vessel obstruction, wilting, and defoliation eventually lead to host plant death^53^.

The management of tomato wilt disease caused by *Fusarium oxysporium f.sp. lycopersici* through chemical fungicides presents several challenges, including residual toxicity, environmental pollution, and the development of pathogen resistance to repeatedly used fungicides^4^. In contrast, employing biocontrol agents such as halophilic bacteria effectively mitigates these issues. Numerous microbes, recognized as biocontrol agents such as *Bacillus spp., Pseudomonas spp*., *Streptomyces spp*., and *Trichoderma spp*. have demonstrated significant efficacy against soil-borne pathogens^58^. These agents compete for ecological substrates by producing antibiotics, hydrogen cyanide, releasing siderophores, and secreting enzymes that lyse fungal cell walls, thereby functioning as effective biocontrol agents^49^. Additionally, they activate induced systemic resistance (ISR) across various crops against multiple diseases, utilizing signaling pathways involving jasmonic acid (JA), ethylene (ET), and salicylic acid (SA)^4^. The antagonistic effect of isolate QSLA1 against *Fusarium* is likely attributed to one or more of these mechanisms.

Isolate QSLA1 has been confirmed to produce IAA (0.15 µg/ml) and possesses nitrogen-fixing capabilities, contributing to improved plant growth, notably in shoot height and root length. Additionally, this strain can produce lipase, protease, and chitinase enzymes recognized for their antimicrobial^30^ and antifungal^13^ properties. GC–MS analysis indicates that strain QSLA1 can synthesize metabolites such as Desulphosinigrin, Undeca-2, 4, 6, 8, 10-pentaenal, 11-(2-furyl)-oxime, and Strychane, 1-acetyl-20α-hydroxy-16-methylene, all of which are acknowledged as potent antifungal and antimicrobial constituents^28^.

Strains from the *Bacillus, Virgibacillus,* and *Halomonas* genera, were isolated from saline habitats in northeastern Algeria, demonstrate remarkable activity against pathogenic fungi including *Botrytis cinerea, Fusarium oxysporium, F. verticillioides,* and *Phytophthora capsici*^35^. Furthermore, tomato plants treated with moderately halophilic bacteria *Halomonas* sp. K2-5, isolated from various Tunisian Sebkhas (hypersaline soils), exhibited reduced stem canker lesions under greenhouse conditions^63^.

## Conclusion

This study demonstrates the potential of halophilic bacterial strains isolated from solar saltern ponds, attached to Qarun Lake, Fayoum governorate, Egypt, to promote plant growth and resist tomato wilt disease under saline conditions. The three isolated strains showed promising results in producing plant growth-promoting substances and one strain exhibited significant antagonistic activity against _*Fusarium*_ fungus, a pathogen affecting tomato seedlings. These findings suggest that these bacterial strains could be used as biofertilizers or biocontrol agents to improve crop productivity and resistance to disease in salt-affected soils.

## Data Availability

Sequence data that support the findings of this study have been deposited in NCBI with the primary accession code OP442496, OP442497, &OP442498.
